# Supplementary material for: Predictive Value of MUC5AC Signature in Pancreatic Ductal Adenocarcinoma: A Hypothesis Based on Preclinical Evidence
Source: Int J Mol Sci. 2023 Apr 30;24(9):8087. doi: 10.3390/ijms24098087 (PMC10178741; doi:10.3390/ijms24098087)
Supplement: Supplementary file 1 [file ijms-24-08087-s001.zip › ijms-2371685-supplementary.pdf]

Supplementary Table S1: Baseline characteristics of pancreatic cell lines

Pancreatic cell line	Source	Metastasis	Doubling time (in hours)	Differentiation	Protein markers
MIA PaCa 2 (M) [79]	Primary Tumor	Nd	40	Poor	No CEA
PANC-1 [80]		Y	52	Poor	No CEA
BxPC-3[81]		N	48-60	Moderate-poor	CEA, hp-CAA; hp-SA and traces of mucin
SU86.86[82]	Liver metastatic lesion	Y	77	Moderate-poor	CEA
CAPAN-1[83]		Y	41	Well	Mucin, CEA\LDH and Beta-microglobulin
COLO357 (CO) [84]	Lymph node	Y	21	Well	Beta-HCG. CEA, mucin-producing
T3M4[85]		Y	31	Well	CEA
AsPc-1[86]	Ascites fluid	Y	38-40	Poor	Abundant mucin and CEA
SW1990 [87]	Splenic metastases	Y	36-40	Moderate	CEA, LDH producing

Abbreviations: hp-CAA -human pancreas cancer-associated antigen, hp-SA human pancreas-specific antigen, Y-yes and N-no; CEA – carcinoembryonic antibody; LDH – lactate dehydrogenase

Supplementary Table S2: Studies used for analyzing the impact of MUC5AC on drug sensitivity

mRNA	Protein expression	PC cell line	Michalski et al. [64]	Fujita et al. [76]	Hofmann et al. [65]
H	H	COLO357 (CO.)	✓		✓
H	H	SW1990 (SW)		✓	
M	M	BXCP3 (B)	✓	✓	
M	L	ASPC-1 (A)	✓	✓	
No	No	Mia Pa Ca (M)	✓	✓	✓
M	No	PANC-1 (P)	✓	✓	✓
L	No	CAPAN-1 (C)	✓	✓	
H	L	T3M4	✓		
Chemotherapy agents studied in the study			5FU, Gem, OX, Cis, Paclitaxel, Mitomycin, GemOx, Gem/MMC	Gem, 5FU	Irinotecan

H-high; L-low; M-moderate expression; Gem-gemcitabine; Ox-oxaliplatin; MMC-mitomycin; Cis-cisplatin

Supplemental Figure S1. MUC5AC's mechanism of action in pancreatic ductal adenocarcinoma

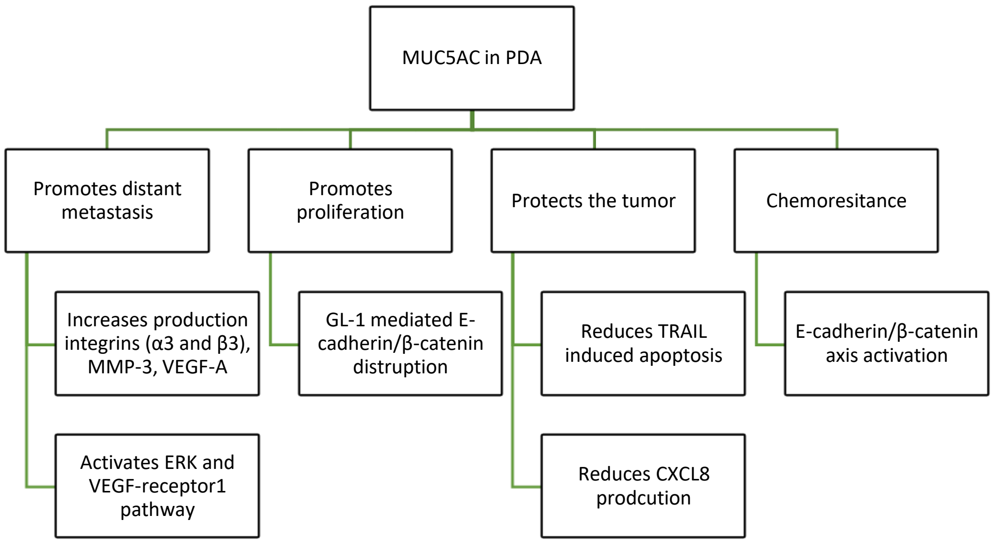

MMP-3, matrix metalloproteinase (MMP) -3; VEGF-A, vascular endothelial growth factor (VEGF) –A; ERK, extracellular-signal-regulated kinase; TRAIL, TNF-related apoptosis-inducing ligand
